# *flrA*, *flrB* and *flrC* regulate adhesion by controlling the expression of critical virulence genes in *Vibrio alginolyticus*

**DOI:** 10.1038/emi.2016.82

**Published:** 2016-08-03

**Authors:** Gang Luo, Lixing Huang, Yongquan Su, Yingxue Qin, Xiaojin Xu, Lingmin Zhao, Qingpi Yan

**Affiliations:** 1 Fisheries College, Key Laboratory of Healthy Mariculture for the East China Sea, Ministry of Agriculture, Jimei University, Xiamen, Fujian 361021, China; 2 College of Ocean and Earth Sciences, Xiamen University, Xiamen, Fujian 361102, China; 3 State Key Laboratory of Large Yellow Croaker Breeding, Ningde, Fujian 352000, China

**Keywords:** adhesion, *flrA*, *flrB*, *flrC*, RNAi, *Vibrio alginolyticus*

## Abstract

Adhesion is an important virulence trait of *Vibrio alginolyticus*. Bacterial adhesion is influenced by environmental conditions; however, the molecular mechanism underlying this effect remains unknown. The expression levels of *flrA*, *flrB* and *flrC* were significantly downregulated in adhesion-deficient *V. alginolyticus* strains cultured under Cu^2+^, Pb^2+^, Hg^2+^ and low-pH stresses. Silencing these genes led to deficiencies in adhesion, motility, flagellar assembly, biofilm formation and exopolysaccharide (EPS) production. The expression levels of *fliA, flgH, fliS, fliD, cheR, cheV* and V12G01_22158 (Gene ID) were significantly downregulated in all of the RNAi groups, whereas the expression levels of *toxT, ctxB, acfA, hlyA* and *tlh* were upregulated in *flrA*- and *flrC*-silenced groups. These genes play a key role in the virulence mechanisms of most pathogenic *Vibrio* species. Furthermore, the expression of *flrA*, *flrB* and *flrC* was significantly influenced by temperature, salinity, starvation and pH. These results indicate that (1) *flrA*, *flrB* and *flrC* are important for *V. alginolyticus* adhesion; (2) *flrA*, *flrB* and *flrC* significantly influence bacterial adhesion, motility, biofilm formation and EPS production by controlling expression of key genes involved in those phenotypes; and (3) *flrA*, *flrB* and *flrC* regulate adhesion in the natural environment with different temperatures, pH levels, salinities and starvation time.

## INTRODUCTION

*Vibrio alginolyticus* is an important opportunistic pathogen and causes vibriosis, one of the most prevalent diseases in maricultured animals and seafood consumers.^1^ The large yellow croaker (*Pseudosciaena crocea*) is one of the most economically important maricultured fish species in southeast China.^2^ *V. alginolyticus* readily causes vibriosis in cultured *P. crocea*, which results in severe economic losses in the warm-water season.^3^

Bacterial adhesion to host surfaces is one of the initial steps in the infection process.^4, 5^ Host mucus is abundantly found on the surface of the skin, gills and gut lining; therefore, it is the first site of interaction between the pathogen and its host.^6^ Thus, research into adhesion of pathogenic bacteria to host mucus has gained much interest^7, 8^ and the genes involved in bacterial adhesion are starting to be explored.^9, 10^ However, to our knowledge, the studies on the genetic regulation of adhesion in pathogenic bacteria conducted thus far are limited, and the genetic regulatory network is still largely unknown.

Bacterial adhesion of pathogenic *V. alginolyticus* to the mucus of *P. crocea* has been studied by our laboratory for several years. The characteristics of *V. alginolyticus* adhesion were found to correlate with its epidemiologic characteristics,^11, 12^ and bacterial adhesion was found to be influenced by environmental factors such as temperature, pH and salinity, among others.^13^ To explore the mechanisms by which environmental factors influence bacterial adhesion, pathogenic *V. alginolyticus* was subcultured under nine different stress conditions. Of these, Cu^2+^, Pb^2+^, Hg^2+^ and low pH were found to reduce the adherence of *V. alginolyticus*, whereas high pH, low or high salinity, and low or high temperature did not significantly affect adhesion.^14^ Next, the adhesion-attenuated culture and a control culture were subjected to RNA-seq.^14^ The RNA-seq results indicated a role for the flagellar assembly genes and three noncoding RNAs, and these were further studied to determine their role in bacterial adhesion.^3, 15^

The flagellum has been shown to be involved in bacterial adhesion and invasion of host cells, as it is used in motility, chemotaxis and as an adhesin.^16^ The synthesis and assembly of the flagellum is a tightly regulated process.^17^ *flrA*, *rpoN*, *flrB* and *flrC* are part of a two-component system^18^ that is considered a key prerequisite for bacterial pathogenesis.^19^ The histone-like nucleoid-structuring protein and the general stress-response regulator RpoS could enhance the transcription of *flrA* and *rpoN*.^20^ Both of FlrA and RpoN-bound RNA polymerase could activate the transcription of class II flagellar genes.^21^ The *flrA* transcription start is 68 bp upstream from the *flrA* translation start site, and DNA sequence upstream of the transcription start contains a σ^70^ promoter sequence (TTGACA-N14-TGGCACTTT).^22^ FlrA could directly bind to the *flrBC* promoter. The binding site is 73–53 bp from the transcription start site (consisted of an inverted repeat sequence of ATTG(A/G)C), which is 24 bp upstream of the translation start site of the *flrBC* operon.^23^ FlrB and FlrC could activate the transcription of class III genes.^21^ *flrA*, *flrB* and *flrC* were first identified in *V. cholerae* and shown to be required for flagellar synthesis.^21^ Since then, only a few related studies were performed and mainly focused on the role of *flrA*, *flrB* and *flrC* in flagellar synthesis.^17, 24^ However, the role they and their downstream genes have in the virulence mechanisms of most pathogens is poorly understood and remains to be elucidated.

The results of RNA-seq by our laboratory showed that *flrA*, *flrB* and *flrC* were significantly downregulated under several stress conditions (Cu^2+^, Pb^2+^, Hg^2+^ and low pH). As *V. alginolyticus* adhesion was also significantly inhibited by these four stress conditions, these genes were hypothesized to have a role in bacterial adhesion. Here, we examine the genes that contribute to adhesion of pathogenic *V. alginolyticus*. The purpose of our study was to investigate (i) the relationship between *flrA*, *flrB*, *flrC* and *V. alginolyticus* adhesion; (ii) how these genes affect adhesion; and (iii) whether these genes participate in the regulation of adhesion in natural conditions.

## MATERIALS AND METHODS

### Bacterial strain and culture conditions

Pathogenic *V. alginolyticus* (ND-01) was isolated from a spontaneously infected *P. crocea* and previously identified as pathogenic by subsequent artificial infection.^25^ Bacteria were stored in physiological saline with 10% glycerol at −80 °C. Bacteria were grown in LB_20_ medium (Luria-Bertani medium plus 2% NaCl)^26^ with shaking at 220 rpm.

RNA sequencing (RNA-Seq) results were confirmed by quantitative reverse transcriptase real-time polymerase chain reaction (qRT-PCR). *V. alginolyticus* was stressed with 50 mg/L Cu^2+^, 100 mg/L Pb^2+^, 50 mg/L Hg^2+^ or low pH (HCl was used to lower the pH to 5). As a control, *V. alginolyticus* was also incubated under normal conditions (LB_20_ broth with 2% NaCl, pH=7). Each treatment consisted of six independent biological replicates (three technical replicates within each).

To investigate the effects of different temperatures, *V. alginolyticus* was cultured in LB_20_ broth (supplemented with 2% NaCl, pH=7) at 4°C, 15°C, 28°C, 37°C and 44 °C. To investigate the effects of different pH levels, *V. alginolyticus* was cultured in LB_20_ broth (supplemented with 2% NaCl) adjusted to a pH of 5, 6, 7, 8 or 9.^11^ To investigate the effects of different salinities, *V. alginolyticus* was cultured in LB_20_ broth (pH=7) with 0.8%, 1.5%, 2.5%, 3.5% or 4.5% NaCl.^11^ To investigate the effects of starvation, *V. alginolyticus* was cultured in normal phosphate-buffered saline (PBS) at 28 °C for one, three, five and seven days.^27^ Each treatment consisted of six independent biological replicates (three technical replicates within each). After harvesting and re-suspending, RNA was extracted from the bacteria, and reverse transcription and qRT-PCR were performed.

*Escherichia coli* SM10 was purchased from TransGen Biotech (Beijing, China). Cultures were grown in LB_20_ broth (220 rpm, 37 °C) or on LB_20_ plates (with 1.8% agar, 37 °C).

### Functional classification and enrichment analysis of differentially expressed genes

To annotate identified differentially expressed genes (DEGs), the Blast2GO program was used to obtain Gene Ontology (GO) annotations for each unigene. After obtaining the GO annotation of each unigene, the WEGO software (BGI, Shenzhen, China) was used to determine GO functional categories of all unigenes and to illustrate the species layout of unigene functions in a macroscopic view. The Bonferroni correction was used to calculate the *P-*value. Adjusted *P*⩽0.05 was used as a threshold. GO terms satisfying this criterion were defined as notably enriched GO terms from the DEGs.

Kyoto encyclopedia of genes and genomes (KEGG) pathway annotations were obtained using the Blastall software (NCBI, Bethesda, MD, USA; http://www.ncbi.nlm.nih.gov/BLAST/) and the KEGG (http://www.genome.jp/kegg/) database. *Q*-values were defined as the false discovery rate analog of the *P*-value. Pathways with *Q*⩽0.05 were deemed significantly enriched in DEGs.

### Transient gene silencing

Negative control short interfering RNA (siRNA) and treatment siRNA sequences (targeting at 136 bp–155 bp downstream from the *flrA* translation start site, 728 bp–747 bp downstream from the *flrB* translation start site and 935 bp–954 bp downstream from the *flrC* translation start site) were chemically synthesized by GenePharma Co. Ltd (Shanghai, China) and are listed in Supplementary Table S1.

Electrotransformation of bacteria was performed following the method of Lancashire *et al.*^28^ Overnight cultures of *V. alginolyticus* were inoculated into fresh LB_20_ broth (1:100 v/v) and incubated until the OD_600_ reached 0.3–0.5. Bacterial cells were harvested by centrifugation at 4000 *g* for 10 min at 4 °C, thoroughly washed twice with ice-cold sterile water at 4 °C and subsequently washed once in ice-cold 10% washing buffer (10% redistilled glycerol, 90% sterile water, v/v). Finally, the competent cells were re-suspended in 1 mL ice-cold 10% glycerol and stored at −80 °C for a short time.

*V. alginolyticus*-competent cells were electroporated using a Bio-Rad MicroPulser (Bio-Rad Laboratories Inc., Hercules, CA, USA) following the method of Huang *et al.*^15^ Two microliters of 20 μM siRNA was vigorously mixed into 100 μL of electrocompetent cells. After incubating on ice for 30 min, the mixture of cells/siRNA was transferred into an electroporation cuvette and electroporated at 1.8 kV for 6 ms. Then, 900 μL of room temperature LB_20_ medium was immediately added, and the cells were shaken for 1 h, 6 h, 12 h and 24 h at 28 °C for RNA extraction and qRT-PCR.

### Stable gene silencing

The stable gene-silencing assay for *V. alginolyticus* was implemented based on Darsigny *et al.*^29^ and Choi and Schweizer.^30^ Overnight cultures of *V. alginolyticus* were collected by centrifugation and treated as described in the transient gene-silencing section. Three short hairpin RNA sequences targeting at 136 bp–155 bp downstream from the *flrA* translation start site, 728 bp–747 bp downstream from the *flrB* translation start site and 935 bp–954 bp downstream from the *flrC* translation start site were designed by Shanghai Generay Biotech Co., Ltd (Shanghai, China; Supplementary Table S2). The annealed oligonucleotides were ligated with pACYC184 vectors, which were harvested from *Bam*HI and *Sph*I double digestion via T4 DNA ligase (TaKaRa, Kusatsu, Japan) according to the manufacturer's recommendations. Competent *E. coli* SM10 cells were transformed with the recombinant pACYC184 vectors via heat-shock and then transferred by conjugation from *E. coli* SM10 to *V. alginolyticus*. The empty plasmid pACYC184 was also transferred into cells and used as the control strain. The stable silenced clones were screened using LB_20_ medium containing chloramphenicol (34 μg/mL).

### Mucus preparation

*P. crocea* care and use were conducted in strict accordance with the recommendations in the ‘Guide for the Care and Use of Laboratory Animals' set by the National Institutes of Health. The protocol was approved by the Animal Ethics Committee of Xiamen University (Acceptance NO XMULAC20120030).

Healthy *P. crocea* were obtained from marine cultured cages in the city of Ningde (Fujian, China) and were used for mucus preparation as previously described.^31^ After careful washing in PBS (0.01 mol/L pH 7.2), the skin mucus was collected by gently scraping the skin surface using a soft rubber spatula and was then homogenized in PBS. The homogenate was centrifuged twice (20 000*g*, 4 °C, 30 min), and the supernatant was filtered through 0.45-μM and 0.22-μM pore-size filters. Finally, PBS was added to each mucus sample to obtain a final concentration of 1 mg protein/mL, as determined by a Bradford assay.^32^

### *In vitro* adhesion assay

The bacterial adhesion assay was carried out as described in our previous study.^33^ *P. crocea* mucus (50 μL) was evenly coated onto a 22-mm^2^ area of a glass slide area and fixed by methanol for 20 min. One milliliter of a bacterial suspension (10^8^ colony-forming unit/mL) was spotted onto a mucus-coated glass slide. Then, the glass slides were placed in a humidified chamber, incubated at 25 °C for 2 h and then washed five times in PBS. Finally, the bacteria were fixed with 4% methanol for 30 min, stained with crystal violet for 3 min and counted under a microscope (× 1000). Three independent biological replicates (three technical replicates within each) were performed per group, and 20 random fields within each technical replicate were chosen to count. For the transient silencing, wild *V. alginolyticus* containing negative control siRNA (non-targeting) was used as a control group. For the stable silencing, wild *V. alginolyticus* containing empty plasmid pACYC184 was used as a control group.

### RNA extraction and reverse transcription

Total bacterial RNA was extracted with TRIzol reagent (Invitrogen, Carlsbad, CA, USA) following the manufacturer's instructions. First-strand complimentary DNA (cDNA) synthesis was performed with a RevertAid Mu-MLV cDNA Synthesis Kit (TransGen Biotech) following the manufacturer's instructions.

### qRT-PCR

The expression levels of genes were determined by qRT-PCR using QuantStudio 6 Flex (Life Technologies, Grand Island, NY, USA). Expression levels were normalized to 16S rRNA and then calculated using the 2^−ΔΔCt^ method; primer sequences are listed in Supplementary Table S3.

### Transmission electron microscope observation

Wild-type and stably silenced *V. alginolyticus* were cultured in LB_20_ broth at 28 °C for 16 h. Then, the culture was mounted onto formvar-coated copper grids by floating the grids on drops of sample for 1 min. The grids were washed three times in sterile deionized water to remove the medium. The cells were negatively stained by floating the grids on a drop of 1% phosphotungstic acid and then viewed using a transmission electron microscope (Philips, Tecnai F20, Amsterdam, Holland). More than 20 fields of view were randomly selected in each group. The length and diameter of flagella were measured with the Osiris 4.0 software (Geneva, Switzerland) from images (*n*=6 cells per condition).

### Soft agar plate motility assay

The soft agar method was used to assay the motility of *V. alginolyticus* strains. Briefly, fresh overnight cultures were diluted to an OD_660_ of 0.03 in LB_20_. The cell suspension (1 μL/drop) was dropped onto the center of semisolid agar plates (LB_20_ broth+0.5% agar) and the plates were allowed to incubate for 20 h at 28 °C before measuring the colony diameters; three independent biological replicates (three technical replicates within each) were performed per group.

### Biofilm assay

The biofilm assay for *V. alginolyticus* was implemented based on Lee *et al.*^34^ *V. alginolyticus* strains were grown (overnight, 28 °C) in LB_20_ and then adjusted to the same OD_600_. Bacterial culture (50 μL) was added to 150 μL of LB_20_ per well of a 96-well plate and then incubated at 28 °C for 24 h. Wells were rinsed with sterile PBS, incubated (30 min) with 200 μL crystal violet (1%), washed with sterile PBS and air-dried. The stained biofilm was solubilized with 200 μL 33% acetic acid and then quantitated by measuring OD_570_; three independent biological replicates (three technical replicates within each) were performed per group.

### Quantification of exopolysaccharide

Purification and quantification of exopolysaccharide (EPS) were performed using a slight modification of the methods of Acosta *et al.*^35^ and Kimmel *et al.*,^36^ respectively. Overnight cultures of *V. alginolyticus* were boiled (10 min) and centrifuged (12 000 rpm, 20 min) to remove cells. Trichloroacetic acid (10% final concentration) was added to precipitate proteins and polypeptides. After centrifugation, a filter membrane (0.45 μM) was used to filter the supernatant. Ice-cold ethanol (1 volume) was added to the supernatant fluid, and the coarse EPS was precipitated. After centrifugation, it was re-suspended in sterile distilled water (1/10th the original volume) and dialyzed in dialysis tubing. Phenol (0.3 mL, 6%) was added to the dialyzed EPS (0.5 mL), and then 1.5 mL concentrated sulfuric acid was quickly added and the mixture was shaken. This solution was heated for 15 min in a 100 °C water bath, cooled in cold water and finally quantitated by measuring OD_490_; three independent biological replicates (three technical replicates within each) were performed per group.

### Hemolysis assay

Hemolysis assays were carried out as described by Tsou and Zhu.^37^ Rabbit blood (Ping Rui Biotechnology Co., Ltd, Beijing, China) was prepared by washing three times with PBS. Washed rabbit blood (5 μL) was added to 245 μL of culture supernatants and incubated at 37 °C for 1 h under the relatively mild shaking conditions. After incubation, samples were centrifuged, and the released hemoglobin was measured by OD_540_. The percentage of total hemolysis was calculated by comparing the OD_540_ of the samples with positive (100% lysis by 1% Triton X-100) and negative controls.

### Data processing

The data are presented as the mean±SD. Statistical analysis was performed by one-way analysis of variance with Dunnett's test using the SPSS 13.0 software (Chicago, IL, USA). *P*-value<0.05 were considered statistically significant.

## RESULTS

### RNA-seq screening and validation for DEGs

The RNA-seq results for *V. alginolyticus* cultured under Cu^2+^, Pb^2+^, Hg^2+^ and low-pH conditions (*V. alginolyticus* cultured under normal condition was used as a control group) have been deposited in the NCBI Sequence Read Archive (Accession NO SRP049226). RNA-seq and DEG analysis finally yielded 1637, 1085, 846 and 1791 DEGs in the Cu-, Pb-, Hg- and low-pH-treated groups compared with the control group, respectively. Analysis of GO categories showed that the functional distribution of the DEGs from each stressed group was similar. In the libraries, most of the corresponding biological process genes were involved in cellular processes, metabolic processes, establishment of localization and localization. Most of the cellular component genes encoded proteins associated with cell, cell part, membrane and membrane part; moreover, most of the molecular function genes were associated with binding and catalytic activity. Using KEGG, DEGs were assigned to 164 KEGG pathways. Those pathways with the greatest representation by DEGs were ‘ABC transporter system', ‘Two-component system', ‘Glyoxylate and dicarboxylate metabolism' and ‘Flagellar assembly'. These annotations provide a substantial resource for investigating specific processes, functions and pathways during bacteria adherence. Among the 164 KEGG pathways, some pathways are known to be closely related to adhesion, for example, ‘Two-component system', ‘Bacterial chemotaxis' and ‘Flagellar assembly'. In the present research, the Two-component system was selected for further research. GO and KEGG analyses revealed that there are 67 DEGs involved in Two-component systems. Among the DEGs, FlrA, FlrB and FlrC were generally significantly downregulated in all four of the stressed groups (Table 1).

**Figure 1:**
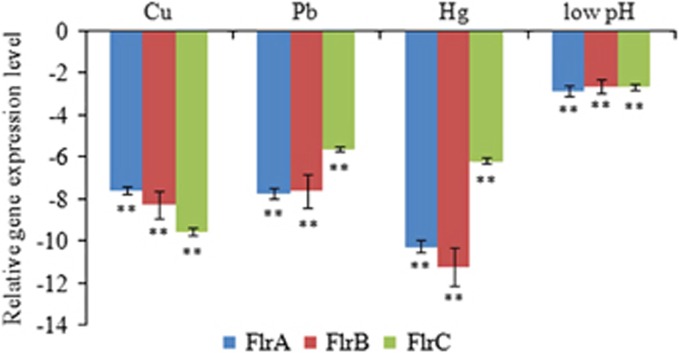
qRT-PCR analysis of the expression of *flrA, flrB* and *flrC* after stress treatments compared with untreated control. The data are presented as the means±SD; each treatment consisted of six independent biological replicates (three technical replicates within each). **P<0.01 compared with control subjects. qRT-PCR, quantitative reverse transcriptase real-time polymerase chain reaction.

To validate the results obtained by RNA-Seq, qRT-PCR was performed on the three genes. The qRT-PCR results were consistent with those in the RNA-Seq results. The Cu^2+^, Pb^2+^, Hg^2+^ and low-pH treatments significantly downregulated the expression of *flrA* (by 7.56-, 7.72-, 10.26- and 2.83-fold, respectively), *flrB* (by 8.27-, 7.61-, 11.22- and 2.60-fold, respectively) and *flrC* (by 9.55-, 5.60-, 6.17- and 2.65-fold, respectively; Figure 1). These data further reinforced the reliability of the RNA-Seq data in this study.

### Effects of transient gene silencing

**Figure 2:**
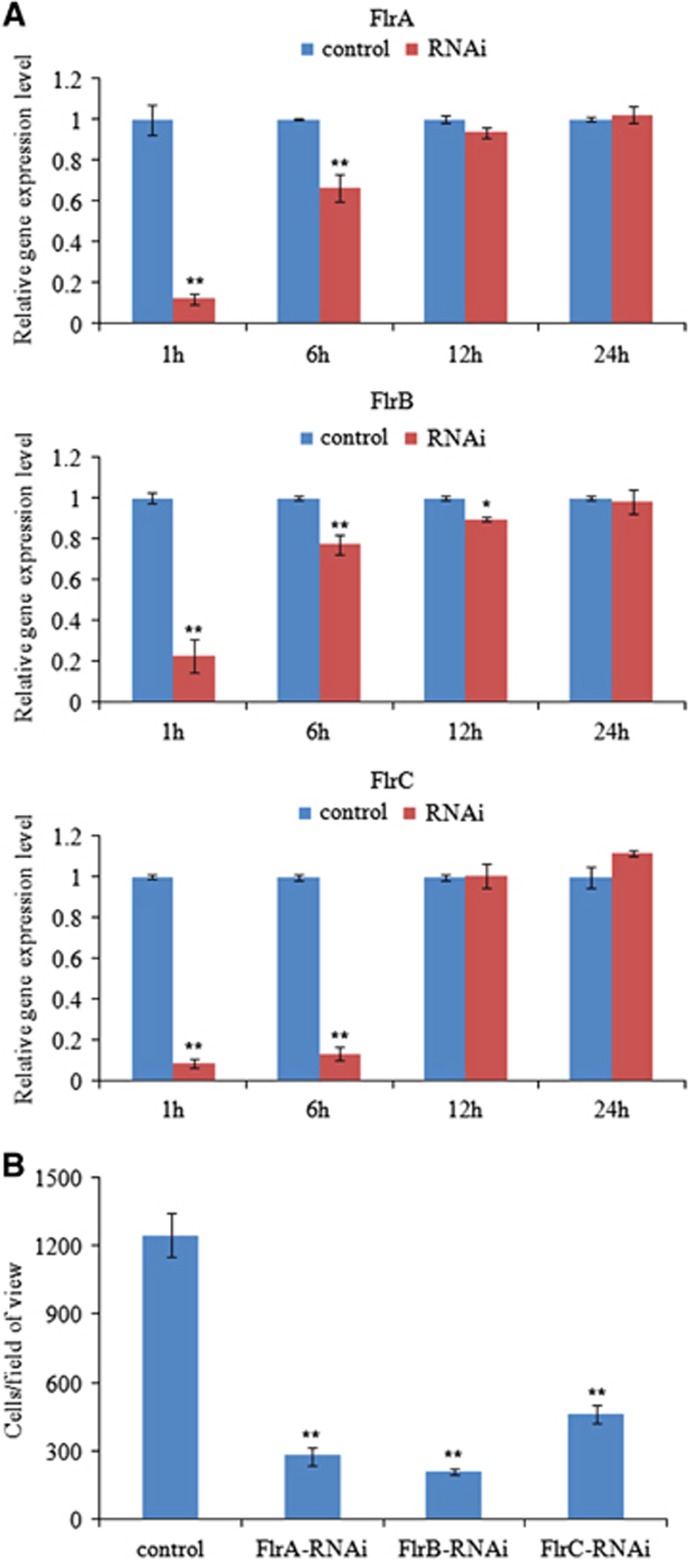
Transient gene silencing reduced the adhesion of *V. alginolyticus*. (**A**) qRT-PCR analysis of the expression of *flrA, flrB* and *flrC* after transient gene silencing at 1 h, 6 h, 12 h and 24 h compared with the control. The data are presented as the means±SD; each treatment consisted of six independent biological replicates (three technical replicates within each). *P<0.05 and **P<0.01 compared with control subjects. (**B**) The adhesion capacity to mucus of transient silenced *V. alginolyticus* at 2 h. The data are presented as the means±SD; three independent biological replicates (three technical replicates within each) were performed per group. *P<0.05 and **P<0.01 compared with control subjects. qRT-PCR, quantitative reverse transcriptase real-time polymerase chain reaction.

The mRNA levels of the three target genes in *V. alginolyticus* were determined at 1, 6, 12 and 24 h after electrotransformation. Treatment of *V. alginolyticus* with siRNA (Figure 2A) induced significant gene silencing (*P*<0.05) in *V. alginolyticus* show at 1–6 h; however, the silencing effect of each siRNA was not significant at any of the time points after 6 h. The decline in mRNA levels of each target gene revealed that the siRNA worked.

Thus, the *in vitro* assay for *V. alginolyticus* adhesion was performed under normal and RNAi conditions to further verify the silencing effect of siRNA at 2 h after electrotransformation. The number of adherent cells was counted. In the control group, ~1248±96 cells/view adhered to the glass. In contrast, in the *flrA-*, *flrB-* and *flrC*-RNAi groups, ~277±40, 207±12 and 463±38 cells/view adhered to the glass, respectively (Figure 2B).

These data show that the number of adherent cells was significantly lower in the RNAi groups than in the control group. The results of this adhesion assay indicate that RNAi significantly impairs *V. alginolyticus* adhesion to *P. crocea* mucus.

### Effects of stable gene silencing

**Figure 3:**
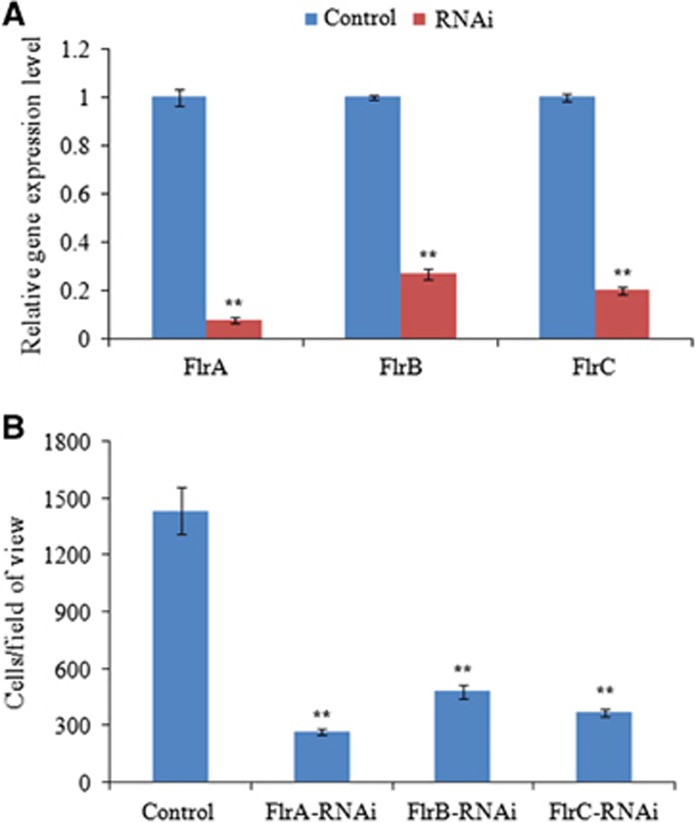
Stable gene silencing reduced the adhesion of *V. alginolyticus*. (**A**) qRT-PCR analysis of the expression of *flrA, flrB* and *flrC* after stable gene silencing compared with the control. The data are presented as the means±SD; six independent biological replicates (three technical replicates within each) were performed per group. **P<0.01 compared with control subjects. (**B**) The adhesion capacity of stably silenced *V. alginolyticus* to mucus. The data are presented as the means±SD; three independent biological replicates (three technical replicates within each) were performed per group. **P<0.01 compared with control subjects. qRT-PCR, quantitative reverse transcriptase real-time polymerase chain reaction.

The mRNA levels of *flrA*, *flrB* and *flrC* were significantly lower (by 12.86-fold, 3.71-fold and 5.03-fold, respectively) in the stably silenced *V. alginolyticus* (Figure 3A). These data indicate that the stable gene silencing was reliable in this study.

The stably silenced *V. alginolyticus* exhibited a significant decrease in adhesion. In the control *V. alginolyticus*, ~1435±122 cells/view adhered to the glass. However, in the *flrA-*, *flrB-* and *flrC*-RNAi *V. alginolyticus*, ~263±15, 480±36 and 367±18 cells/view adhered to the glass, respectively (Figure 3B). The adhesion assay results indicate that stable gene silencing significantly impairs *V. alginolyticus* adhesion to *P. crocea* mucus.

The expression levels of 12 important downstream virulence genes in the stably silenced *V. alginolyticus* are shown in Table 2. Compared with the control group (strain with the empty plasmid pACYC184), the expression levels of *fliA, flgH, fliS, fliD, cheR, cheV* and V12G01_22158 exhibited significant downregulation in all three of the RNAi groups, whereas the expression levels of *toxT, ctxB, acfA, hlyA* and *tlh* exhibited upregulation in the *flrA*- and *flrC*-RNAi groups. These data indicate that the expression levels of the majority of these virulence genes were affected by RNAi of *flrA*, *flrB* and *flrC*. For the upregulated genes, *flrA*-RNAi exhibited a more powerful effect than *flrB*-RNAi and *flrC*-RNAi. Furthermore, the expression of *toxT, ctxB, acfA, hlyA* and *tlh* was unchanged in the *flrB* stably silenced strain compared with the control. For the downregulated genes, *flrB*-RNAi exhibited a more powerful effect than *flrA*-RNAi and *flrC*-RNAi; however, the decrease in *fliD* transcription was most obvious in the *flrA* stably silenced strain.

**Figure 4:**
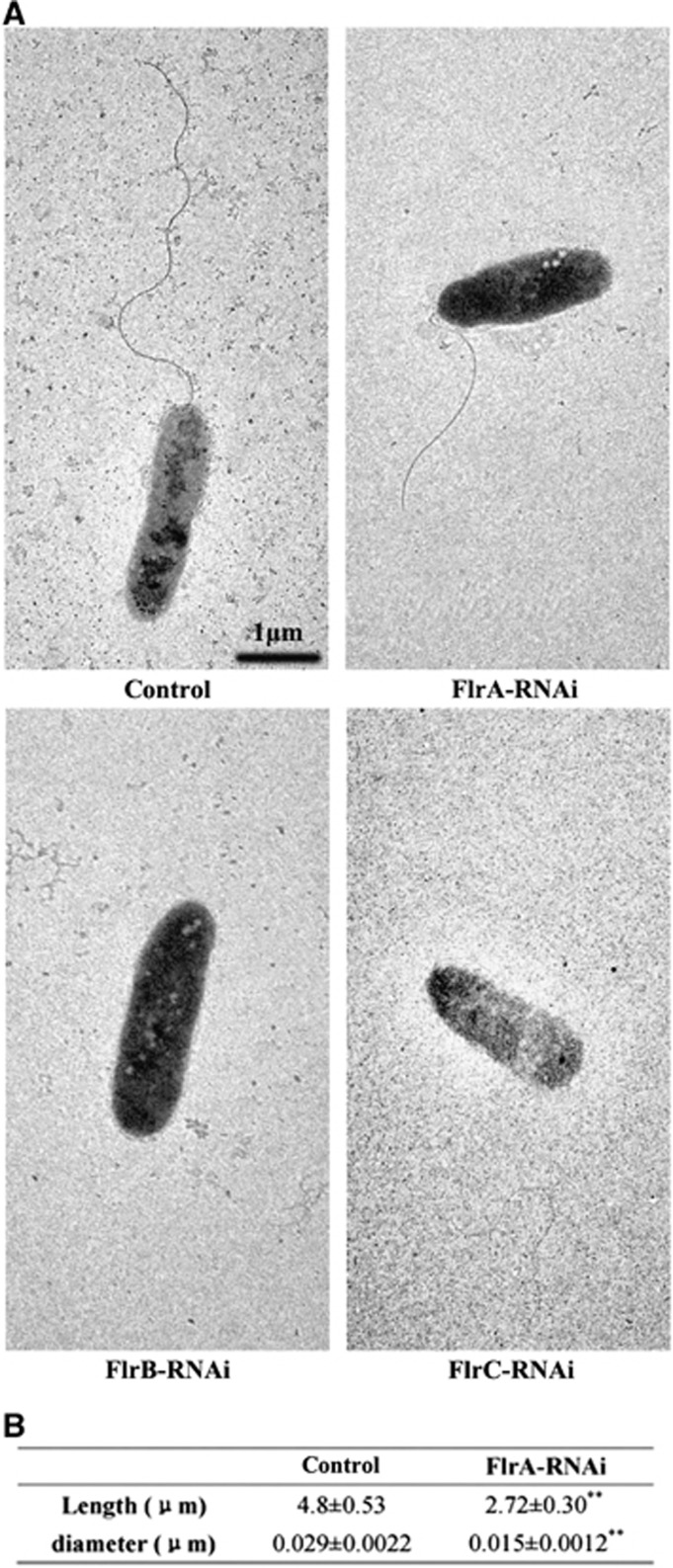
Detection of flagella in stably silenced *V. alginolyticus* strains. (**A**) Transmission electron micrographs of stably silenced *V. alginolyticus* strains. (**B**) Length and diameter of flagella in the control and *flrA*-RNAi strains. The data are presented as the means±SD; **P<0.01 compared with control subjects.

The flagella of stably silenced clones were observed (Figure 4). The data revealed that flagella were present on the control and *flrA*-RNAi strains, whereas the flagella of the *flrA*-RNAi strain were significantly thinner and shorter compared with the control (*P*<0.01). However, no flagella could be observed on the *flrB*-RNAi and *flrC*-RNAi strains.

**Figure 5:**
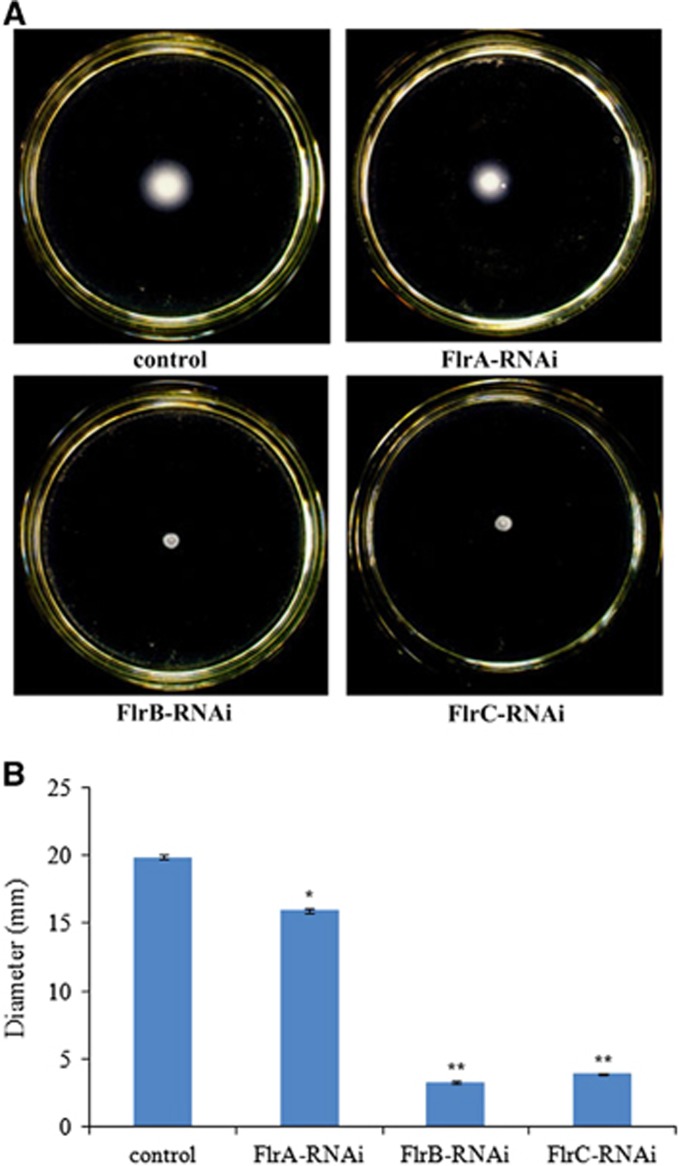
The motility behavior on soft agar plates of stably silenced *V. alginolyticus* strains. (**A**) Typical images of spreading of stably silenced *V. alginolyticus* strains and the control. (**B**) Diameter of the colony of each strain. The data are presented as the means±SD; three independent biological replicates (three technical replicates within each) were performed per group. *P<0.05 and **P<0.01 compared with control subjects.

Figure 5 shows different levels of *V. alginolyticus* motility on soft agar plates. The results indicated that *flrA*-RNAi*, flrB*-RNAi and *flrC*-RNAi resulted in reduced motility of *V. alginolyticus,* whereas *flrB*-RNAi and *flrC*-RNAi were more effective than *flrA*-RNAi.

**Figure 6:**
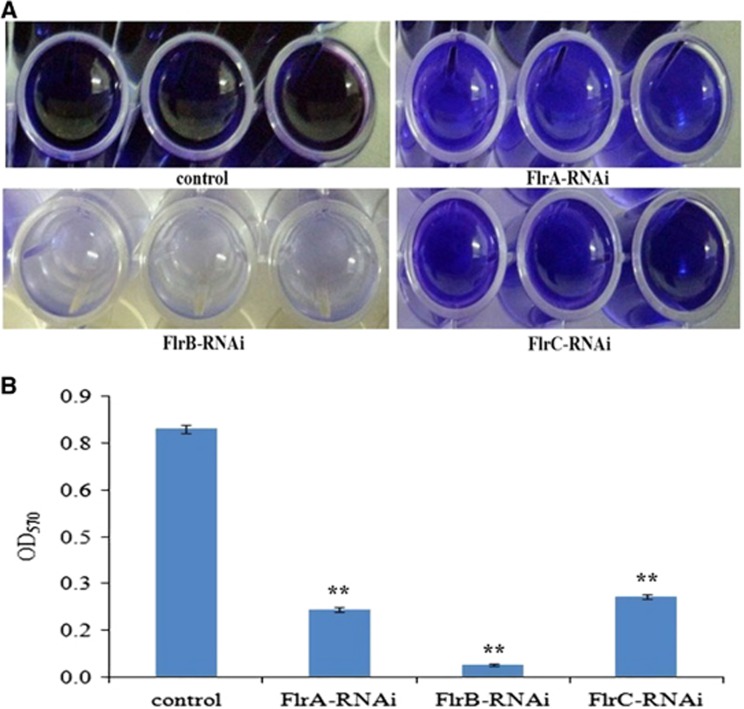
Biofilm formation of stably silenced *V. alginolyticus* strains. (**A**) Typical images of stained biofilm of stably silenced *V. alginolyticus* strains and the control. (**B**) OD_570_ of stained biofilm in the colony of each strain. The data are presented as the means±SD; three independent biological replicates (three technical replicates within each) were performed per group. **P<0.01 compared with control subjects.

Biofilm formation of stably silenced clones was measured at OD_570_ (Figure 6). The data showed that the ability to form biofilms was significantly decreased in *flrA*-RNAi*, flrB*-RNAi and *flrC*-RNAi compared to the wild-type strain, whereas *flrB*-RNAi was more effective than *flrA*-RNAi and *flrC*-RNAi (*P*<0.05).

**Figure 7:**
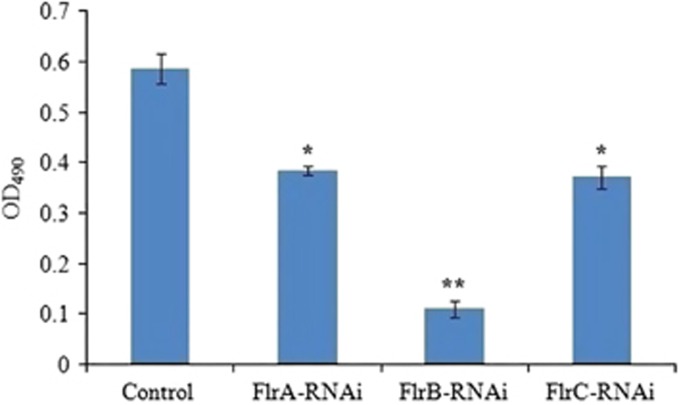
Production of EPS for stable silenced *V. alginolyticus* strains. The data are presented as the means±SD; three independent biological replicates (three technical replicates within each) were performed per group. *P<0.05 and **P<0.01 compared with control subjects. exopolysaccharide, EPS.

Figure 7 shows the production of EPS in different *V. alginolyticus* strains. The results revealed that EPS production of *flrA*-RNAi*, flrB*-RNAi and *flrC*-RNAi was significantly lower than the control, whereas the EPS production of *flrB*-RNAi was the lowest of the three stably silenced strains (*P*<0.05).

**Figure 8:**
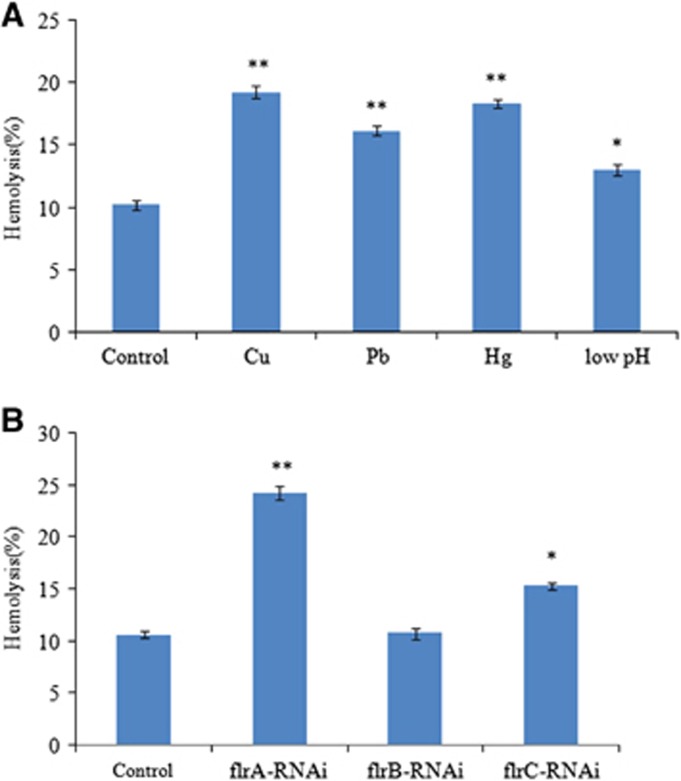
The hemolytic activity of *V. alginolyticus* strains under stress conditions of Cu^2+^, Pb^2+^, Hg^2+^ and low pH (**A**) and after stable gene silencing (**B**). The data are presented as the means±SD; three independent biological replicates (three technical replicates within each) were performed per group. *P<0.05 and **P<0.01 compared with control subjects.

The hemolytic activity of *V. alginolyticus* strains during stress conditions of Cu^2+^, Pb^2+^, Hg^2+^ and low pH, and during stable gene silencing has been detected (Figure 8). The *V. alginolyticus* strains under stress conditions of Cu^2+^, Pb^2+^, Hg^2+^ and low pH showed increased levels of a hemolytic activity against rabbit erythrocytes compared with the *V. alginolyticus* strains under normal condition (Figure 8A). The Figure 8B shows that hemolytic activity was significantly increased in *flrA*-RNAi and *flrC*-RNAi compared with the wild-type strain, and *flrA*-RNAi was more effective than *flrC*-RNAi, whereas hemolytic activity was not significantly increased in *flrB*-RNAi compared with the wild-type strain.

### Effects of different environmental conditions on the expression of *flrA*, *flrB* and *flrC*

**Figure 9:**
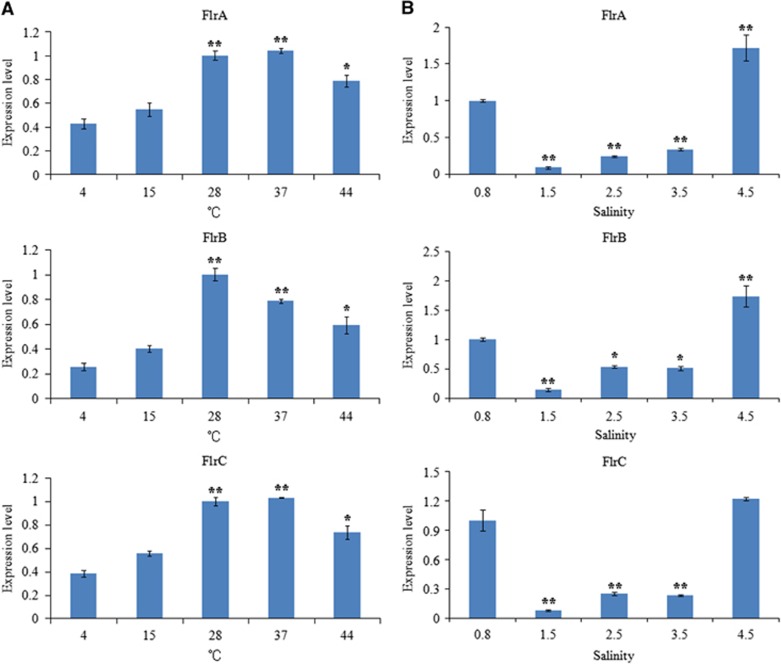
qRT-PCR analysis of the expression of *flrA, flrB* and *flrC* in the *V. alginolyticus* under different temperatures (**A**) and salinities (**B**). The data are presented as the means±SD; each treatment consisted of six independent biological replicates (three technical replicates within each). *P<0.05 and **P<0.01 compared with control subjects. qRT-PCR, quantitative reverse transcriptase real-time polymerase chain reaction.

**Figure 10:**
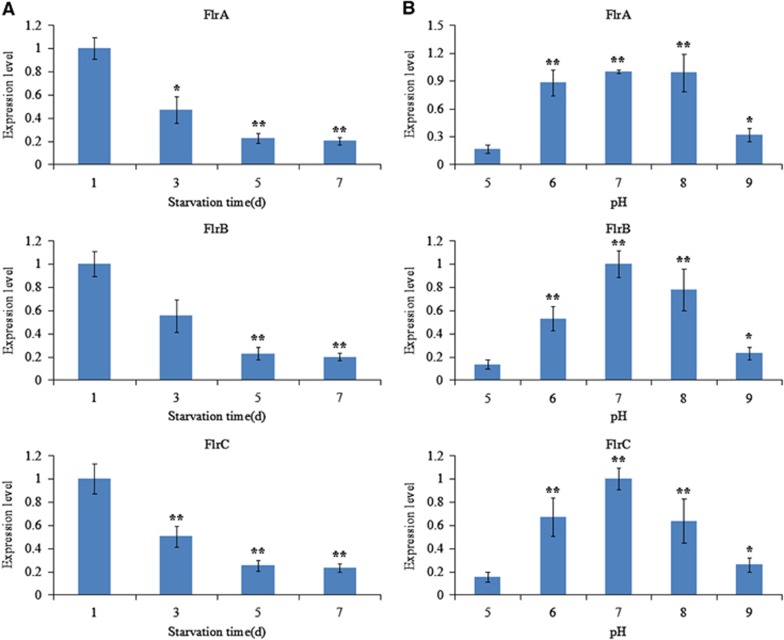
qRT-PCR analysis of the expression of *flrA, flrB* and *flrC* in the *V. alginolyticus* under different starvation time (**A**) and various pH values (**B**). The data are presented as the means±SD; each treatment consisted of six independent biological replicates (three technical replicates within each). *P<0.05 and **P<0.01 compared with control subjects. qRT-PCR, quantitative reverse transcriptase real-time polymerase chain reaction.

The expression of *flrA*, *flrB* and *flrC* under different environmental conditions was measured by qRT-PCR, and the results are shown in Figures 9 and 10.

The expression levels of *flrA*, *flrB* and *flrC* were essentially consistent under the different environmental conditions tested. *flrA*, *flrB* and *flrC* exhibited in part significant variation at different temperatures, with the highest expression levels of all the three genes occurring at 28 °C (Figure 9A). *flrA*, *flrB* and *flrC* expression levels were the lowest at 1.5% salinity; however, they later rose with increasing salinity (Figure 9B). Increasing starvation time also tended to reduce the expression levels of *flrA*, *flrB* and *flrC* (Figure 10A). Expression of *flrA*, *flrB* and *flrC* was significantly affected by pH, and the highest expression levels of all the three genes occurred at pH 7 (Figure 10B). These data indicate that *flrA*, *flrB* and *flrC* may participate in regulation of adhesion in the natural environment.

## DISCUSSION

Bacterial adhesion is ultimately regulated by genes.^38^ Several adhesion-related genes have been identified in *V. alginolyticus*,^3^ *Pseudomonas putida*,^39^ *P. aeruginosa*,^39^ *Candida albicans*,^40^ *E. coli*^41^ and *Erysipelothrix rhusiopathiae*.^42^ However, more details about the mechanism have not been found.

In our previous research,^14^ *V. alginolyticus* adhesion was significantly reduced by Cu^2+^, Pb^2+^, Hg^2+^ and low pH. What's more, *flrA, flrB* and *flrC* were generally downregulated under the four stress conditions tested. The flagellum has been shown to be involved in bacterial adhesion,^16^ whereas *flrA*, *flrB* and *flrC* are required for the synthesis and assembly of the flagellum.^21^ We do not think this is a coincidence. Therefore, we hypothesized a connection between them. In the present study, RNAi-mediated silencing of these three genes reduced bacterial adhesion. These results demonstrate that *flrA, flrB* and *flrC* have a key role in *V. alginolyticus* adhesion.

Bacterial adhesion has been demonstrated to be affected by bacterial motility^19^ and chemotaxis.^16^ Indeed, bacterial motion and chemotaxis rely on the synthesis and assembly of the flagellum.^43^ Transcription of the polar flagellar genes in *V. cholerae* has been categorized into four gene classes.^44^ In *V. cholerae*, the sole class I gene identified so far is *flrA*, which encodes a σ^54^ (RpoN)-dependent transcriptional activator.^17^ FlrA and RpoN-bound RNA polymerase could activate the transcription of the class II flagellar genes.^21^ Of the Class II genes, *fliA* (σ^28^), *flrB* and *flrC* encode regulatory factors. *flrB* undergoes autophosphorylation and then activates *flrC* by transferring a phosphate to the conserved aspartate-54 (D54) residue at the amino terminus of *flrC* (*flrC*-P), allowing it to activate the σ^54^-dependent transcription of class III genes, *fliA* activates the transcription of class IV genes. Of the Class III genes, the *flgH, cheR, fliD* and *fliS* encode the L ring, methyltransferase, filament cap and flagellin chaperone, respectively.^45^ Of class IV genes, *cheV* encodes the chemotaxis signal transduction protein.^17^ *fliA, flgH, fliS, fliD, cheR and cheV* were dramatically decreased in the three stably silenced *V. alginolyticus* strains (Table 2). Nevertheless, it is hard to draw a common conclusion for the regulatory mechanism from the presented experimental results. Interestingly, the downregulation of *fliA, flgH, fliS, cheR and cheV* in the *flrB*-RNAi strain was more distinct than in the *flrA*-RNAi and *flrC*-RNAi strains. We also showed that the *flrB*-RNAi has more significant effects on flagella assembly and motility than the *flrA*-RNAi and *flrC*-RNAi strains. In this regard, *flrB* may have a major role in the regulation of flagellar synthesis and chemotaxis. However, the only previously known role the regulator *flrB* had was in flagellar stimulation and transcription of the chemotaxis gene. In addition, in the *flrB*-RNAi strain, downregulation of *fliA* was the most significant compared with *flgH, fliS, fliD, cheR* and *cheV*, suggesting that it has a vital role in the regulatory mechanism. As discussed above, the downregulation of *fliA, flgH, fliS, fliD, cheR* and *cheV* may be mainly concerned with the downregulation of transcription of *flrA*, *flrB* and *flrC*; and then the downregulation of *fliA* induced the downregulation of *cheV*. Thus, the six flagellar genes could be regulated by *flrA*, *flrB* and *flrC*. Downregulation of the expression of these genes may reduce the synthesis and assembly of the MS ring, filament, filament cap, motor/switch complex and chemotaxis protein. In the present study, the flagella of the *flrA*-RNAi strain were significantly thinner and shorter, whereas no flagella could be observed in the *flrB*-RNAi and *flrC*-RNAi strains (Figure 4). The stably silenced strains were less motile compared with the control group (Figure 5). These results were consistent with our speculation. Thus, *flrA*, *flrB* and *flrC* are very important and may affect bacterial adhesion via motility and chemotaxis.

The bacterial adhesion process was also directly mediated by adhesins,^16, 46^ which are widely distributed on the bacterial surface.^47^ *V12G01_22158* is a gene that has recently been identified in pathogenic *V. alginolyticus* and encodes a GGDEF protein^48^ whose domain DGCs catalyze the conversion of two guanosine triphosphate into bis-(3′-5′)-cyclic dimeric guanosine monophosphate, which generally has an essential role as a ubiquitous second messenger in regulating motility, virulence, biofilm formation (associated with adhesins)^49^ and EPS (an adhesin) biogenesis.^50^ These results indicate that *V12G01_22158* may be an important pathogenic gene in *V. alginolyticus*. Our data show that the expression of V12G01_22158 was significantly downregulated in every stably silenced *V. alginolyticus* strains (Table 2), and the three stably silenced strains had reduced biofilm formation and EPS production compared with the wild-type strain (Figures 6 and 7), especially in the *flrB*-RNAi strain. These results indicate that the transcription of V12G01_22158 was controlled by *flrA, flrB* and *flrC*. Thus, the downregulation of V12G01_22158 expression may be one of the reasons for the reduced adhesion of the stably silenced strains.

Genes *acfA, ctxB, hlyA* and *tlh* have a pivotal role in the colonization and pathogenesis of *Vibrio.* The *hlyA* and *tlh* are hemolysin genes.^45^ *toxT* activates transcription of *acfA* and *ctxB*.^51^ It has been shown that many virulence genes are more highly expressed in flagellar regulatory mutants and can enhance *V. cholerae* pathogenesis.^45^ In this study, *flrA*-RNAi resulted in significant upregulation of *toxT, acfA, ctxB, hlyA* and *tlh,* which led to significant increase in hemolytic activity. *flrB*-RNAi exhibited no significant effect on the expression of these five genes and hemolytic activity. *flrC*-RNAi resulted in significant upregulation of *toxT*, *ctxB* and *tlh,* which led to significant increase in hemolytic activity. Enhanced hemolytic activity was also observed for the *V. alginolyticus* strains under stress conditions of Cu^2+^, Pb^2+^, Hg^2+^ and low pH, whereas *flrA* and *flrC* were downregulated by these conditions. Taken together, these results indicated that (i) transcriptions of *acfA, ctxB, hlyA*, *tlh* and *toxT* were negatively regulated by *flrA,* and then the hemolytic activity was repressed by *flrA* through *hlyA* and *tlh;* (ii) transcriptions of *ctxB, tlh* and *toxT* were negatively regulated by *flrC,* and then the hemolytic activity was repressed by *flrC* through *tlh*; (iii) the increase in *acfA* and *ctxB* expression is most likely because of the upregulation of *toxT*, which directly binds and activates *acfA* and *ctxB*.^45^

Our results suggested that expression of the virulent genes *toxT*, *ctxB* and *tlh* was regulated by *flrA* and *flrC*, that the virulence genes *acfA* and *hlyA* were regulated by *flrA*, and that *fliA, flgH, fliS, fliD, cheR, cheV* and *V12G01_22158* were regulated by *flrA*, *flrB* and *flrC*. *flrA*, *flrB* and *flrC* therefore have a significant signaling role in the pathogenicity of flagellation, motility, chemotaxis, adhesion, colonization and cytotoxicity. Moreover, *flrB*-RNAi led to maximum inhibition of genes associated with flagellation, motility, chemotaxis and adhesins, which translated to significant inhibition of adhesion; yet, *flrB*-RNAi did not increase the expression of genes associated with colonization or cytotoxicity. Therefore, *flrB* may be the most important gene among *flrA*, *flrB* and *flrC* from the perspective of disease prevention and treatment.

Many pathogenic bacteria can induce an adaptable response to environmental stimuli, primarily by altering gene expression.^19^ For example, *V. cholera rpoS* mutants are sensitive to environmental conditions (including starvation, high osmolarity and oxidative stresses) when compared with the wild type.^52, 53^ In our present study, the expression levels of *flrA*, *flrB* and *flrC* were found to be sensitive to different temperatures, changes in pH, changes in salinities and increased starvation time (Figures 9 and 10). Our previous research showed that *V. alginolyticus* adhesion was remarkably influenced by those environmental factors.^11^ These results suggested that the changes in expression of *flrA, flrB* and *flrC* may be an important factor influencing adhesion under those environmental conditions and further strengthened the necessity of the present study.

In conclusion, our results suggest that (i) *flrA, flrB* and *flrC* are closely associated with the process of *V. alginolyticus* adhesion; (ii) *flrA*, *flrB* and *flrC* affect adhesion by affecting motility, chemotaxis and adhesins; and (iii) *flrA*, *flrB* and *flrC* regulate adhesion in different natural environments. In addition, our results showed that *flrA*, *flrB* and *flrC* have a significant signaling role in the pathogenicity of flagellation, motility, chemotaxis, adhesion, colonization and cytotoxicity. In this paper, we present for the first time the biochemical and molecular features of the *flrA, flrB* and *flrC* in *V. alginolyticus* and also indicate the pathogenic implications of these genes, which provide insight into prevention and treatment of this and related diseases.

## Figures and Tables

**Table 1:** Fold change of *flrA, flrB and flrC* identified by RNA-seq

	**Cu**	**Pb**	**Hg**	**Low pH**
*flrA*	−5.82	−5.94	−7.89	−2.17
*flrB*	−6.41	−5.90	−8.69	−2.01
*flrC*	−7.52	−4.41	−4.86	−2.08

Abbreviation: RNA sequencing, RNA-seq.

**Table 2:** Expression of 12 important virulence genes after stable gene silencing

	**flrA-RNAi**	**flrB-RNAi**	**flrC-RNAi**
*fliA*	−51.8±0.007^**^	−222.7±0.001^**^	−46.0±0.005^**^
*flgH*	−35.8±0.009^**^	−76.4±0.002^**^	−27.2±0.007^**^
*fliS*	−35.1±0.008^**^	−47.5±0.003^**^	−16.4±0.009^**^
*fliD*	−178.4±0.002^**^	−149.3±0.002^**^	−37.9±0.005^**^
*cheR*	−1.9±0.0007*	−27.5±0.006^**^	−10.2±0.001^**^
*cheV*	−17.7±0.009^**^	−45.3±0.005^**^	−22.4±0.009^**^
V12G01_22158	−47.7±0.007^**^	−196.6±0.001^**^	−36.7±0.007^**^
*toxT*	10.7±0.246^**^	1.2±0.288	11.1±0.258^**^
*ctxB*	3.6±1.123^**^	1.1±0.139	2.0±0.426*
*acfA*	2.9±0.817^**^	1.1±0.130	1.0±0.144
*hlyA*	2.8±0.949^**^	1.0±0.167	1.4±0.256
*tlh*	7.8±0.499^**^	1.2±0.188	2.6±0.477^**^

The data are presented as the means±SD; each treatment consisted of six independent biological replicates (three technical replicates within each). *P<0.05 and **P<0.01 compared with control subjects.
