# Supplementary material for: flrA, flrB and flrC regulate adhesion by controlling the expression of critical virulence genes in Vibrio alginolyticus
Source: Emerg Microbes Infect. 2016 Aug 3;5(8):e85–. doi: 10.1038/emi.2016.82 (PMC5034100; doi:10.1038/emi.2016.82)
Supplement: Supplementary Table 1 [file emi201682x1.pdf]

Supplementary Table S1 siRNA Sequence

Target gene	siRNA for transient gene silence
*flr*A	F: 5' GCGUAUGGUCGUGCUGUAUTT 3'
	R: 5' AUACAGCACGACCAUACGCTT 3'
flrB	F: 5' GGUCAUGAAUGCGAUUCAATT 3'
	R: 5' UUGAAUCGCAUUCAUGACCTT 3'
flrC	F: 5' GCAUCUUGCUGAGCGUCAUTT 3'
	R: 5' AUGACGCUCAGCAAGAUGCTT 3'
Negative control	F: 5'-UUCUCCGAACGUGUCACGUTT-3'
	R: 5'-ACGUGACACGUUCGGAGAATT-3'
