# Supplementary material for: flrA, flrB and flrC regulate adhesion by controlling the expression of critical virulence genes in Vibrio alginolyticus
Source: Emerg Microbes Infect. 2016 Aug 3;5(8):e85–. doi: 10.1038/emi.2016.82 (PMC5034100; doi:10.1038/emi.2016.82)
Supplement: Supplementary Table 2 [file emi201682x2.pdf]

Supplementary Table S2 Oligonucleotides used in producing shRNA for stable gene silencing

Target gene	shRNA sequence for stable gene silence
flrA	F:5'-GATCCGCGTATGGTCGTGCTGTATTTTTCAAGAGAAAATACAGCACGACCATACGCTTTTTTC-3'<br>R:5'-GTACGAAAAAAGCGTATGGTCGTGCTGTATTTTCTCTTGAAAAATACAGCACGACCATACGCG-3'
flrB	F:5'-GATCCGGTCATGAATGCGATTCAATTTTCAAGAGAAATTGAATCGCATTCATGACCTTTTTTC-3'<br>R:5'-GTACGAAAAAAGGTCATGAATGCGATTCAATTTCTCTTGAAAATTGAATCGCATTCATGACCG-3'
flrC	F:5'-GATCCGCATCTTGCTGAGCGTCATTTTTCAAGAGAAAATGACGCTCAGCAAGATGCTTTTTTC-3'<br>R:5'-GTACGAAAAAAGCATCTTGCTGAGCGTCATTTTCTCTTGAAAAATGACGCTCAGCAAGATGCG-3'
