# Supplementary material for: flrA, flrB and flrC regulate adhesion by controlling the expression of critical virulence genes in Vibrio alginolyticus
Source: Emerg Microbes Infect. 2016 Aug 3;5(8):e85–. doi: 10.1038/emi.2016.82 (PMC5034100; doi:10.1038/emi.2016.82)
Supplement: Supplementary Table 3 [file emi201682x3.pdf]

Supplementary Table S3 Primers for q RT-PCR

Gene	Primers for qRT-PCR
flrA	F: 5'AACTCACTGATGGAGCATAACTGGC 3'<br>R: 5'TGGTTGGAACTCTGGGATGTCACT 3'
flrB	F:5'CCAAATCTTCCTCCAGCGACA 3'<br>R: 5'CTACCAACTGGGCAATCGTGAAT 3'
flrC	F: 5' AGCGAAGTTGTTGCGTGTTTT 3'<br>R: 5'-AGGCGGTAATATAAGTCCTCTCTGA 3'
fliA	F: 5'CGGCATCGGGTTATTAAATGCGGT 3'<br>R: 5'ATACGCTGCACTGCGTAAGTGGTA 3'
flgH	F: 5'GTATTGGCGACATTATTACCGTGA 3'<br>R: 5'GAGGTTGCCGTTTGCCAGTA 3'
fliS	F: 5'CTGTCTATCTATGGATGACGGTGGT 3'<br>R: 5'TATGAAACTCTGTTGGGATTTGGTC 3'
fliD	F: 5'TATCACAGGTATTACAGGCAGTATTCG 3'<br>R: 5'CCCAACGCACTCATCAAAGC 3'
cheR	F: 5'GCAGGCTGGACGAACTATCG 3'<br>R: 5'AACTTGGCAATGCTTGTGGA 3'
cheV	F: 5'TCATCGCGGATATTAACCCA 3'<br>R: 5'GATCACATTGTAACCTGAAGAAGCT 3'
V12G01_22158	F:5' AAGAGTCGGAGGGGAGGAGT 3'<br>R:5' GACAATTTTGAATGTGGGAACG 3'
toxT	F: 5'TGATGATCTTGATGCTATGGAGAAA 3'<br>R: 5'TCATCCGATTCGTTCTTAATTCAC 3'
acfA	F: 5'AGCGTATTCGGCGGTATTG 3'<br>R: 5'ACTTGGTAGCTTGCTGATGC 3'
ctxB	F: 5'GGATGAAGGATACCCTGAGGATT 3'<br>R: 5'TGAGGCGTTTTATTATTCCATACACA 3'
hlyA	F: 5'AGCAGCGTGTGGGACAAGAT 3'<br>R: 5'AGGTTGCCGTGATGGATTTC 3'
tlh	F: 5'CACTTGGGAGTGGGCAAAGA 3'<br>R: 5'TGGTTGACACCAAGCGTCTCT 3'
